# Dual Modulation of Adipogenesis and Lipolysis Augments Facial Adipose Tissue Restoration

**DOI:** 10.34133/csbj.0213

**Published:** 2026-09-14

**Authors:** Nayere Taebnia, Magdalena Murawska, Aurino M. Kemas, Hongda Sheng, Yi Wang, Jian Xu, Tingting Zhou, Ivan Galanin, Volker M. Lauschke

**Affiliations:** ^1^ Department of Physiology and Pharmacology and Center for Molecular Medicine, Karolinska Institutet and University Hospital, Stockholm, Sweden.; ^2^ ABS Group Inc., Edmonton, AB, Canada.; ^3^ Pharmaceutical Informatics Institute, College of Pharmaceutical Sciences, Zhejiang University, Hangzhou 310058, China.; ^4^ State Key Laboratory of Chinese Medicine Modernization, Innovation Center of Yangtze River Delta, Zhejiang University, 314102 Jiaxing, China.; ^5^ Department of Dermatology, Hangzhou Third People’s Hospital, Hangzhou, Zhejiang Province 310009, China.; ^6^ Shanghai Hepo Biotechnology Co., Ltd, 201210 Shanghai, China.; ^7^ Adipeau Inc., New York, NY, USA.; ^8^ Dr Margarete Fischer-Bosch Institute of Clinical Pharmacology, Stuttgart, Germany.; ^9^ University of Tübingen, Tübingen, Germany.; ^10^ Department of Pharmacy, the Second Xiangya Hospital, Central South University, Changsha, China.

## Abstract

Aging leads to structural changes in facial adipose tissue, characterized by an imbalance between adipocyte regeneration and hypertrophy. Current therapeutic approaches primarily target individual aspects of these changes, often failing to address the dual nature of facial adipose remodeling. Using a combination of in vivo and ex vivo methods, we present a therapeutic strategy based on *Kaempferia parviflora* root extract (KPRE) rich in polymethoxyflavonoids. Mechanistically, we demonstrate that KPRE sustains preadipocyte proliferation while reducing hypertrophy in mature adipocytes via activation of the transcriptional regulators SOX9 and GATA3 while suppressing PPARγ and SREBP1. To translate these findings toward human application, we conducted a clinical pilot study in 35 participants with topical application of KPRE long-chain fatty acid emulsions for 6 months. By comparing high-resolution stereoscopic imaging and digital 3-dimensional modeling data at baseline and after 3 months and 6 months of follow-up, we show significant volume restoration in atrophic regions and reduction in hypertrophic areas, consistent with the coordinated effects observed in the organotypic adipose culture models. These findings provide a successful example for the rapid translation of molecular insights gained from organotypic human tissue models to human application and provide clinical evidence supporting the potential of KPRE-based formulations for facial adipose tissue rejuvenation.

## Introduction

The youthful face is characterized by a series of unbroken arcs [1]. As the face ages, quantitative and qualitative changes in facial adipose compartments result in cell depletion, compensatory hypertrophy, and a pro-inflammatory microenvironment, impacting this symmetry [2]. Adipocyte depletion and hypertrophy often develop in proximity. In the periorbital region, adipocyte depletion in the tear trough and upper malar fat pad is contrasted by the bulging of orbital fat, creating a noticeable contrast between atrophic and hypertrophic areas [3]. In the perioral region, distinct perioral mounds are positioned near areas of fat depletion and skin laxity, highlighting the contrast between volume loss and localized fat accumulation [4]. Thus, rejuvenation of the aging face often requires a coordinated approach that increases subcutaneous adipose tissue volume in areas of the face characterized by adipocyte depletion while decreasing it in hypertrophic areas. Current approaches to restructure facial adipose tissue volume commonly rely on rhytidectomy to surgically redistribute adipose volume by fat grafting and filler injections. However, while considered generally safe, there remain risks of developing severe hematomas, deep vein thrombosis, scar formation, and visible deformities [5].

Topical administration of compounds and mixtures offers a pharmacological alternative to modulate dermal adipose tissue. Mature adipocytes and preadipocytes are located around the base of hair follicles [6]. These follicles can provide a gateway for the topical delivery of active substances to the dermal adipose tissue [7]. Long-chain fatty acids such as linoleic acid and oleic acid promote the maturation of preadipocytes through PPARγ signaling and the expansion of lipid droplet size in mature adipocytes following CD36 receptor-mediated uptake [8]. Similarly, local activation of PPARγ by topical application of rosiglitazone has been shown to induce localized expansion of dermal white adipose tissue (dWAT) and increased adipogenic marker expression in mice [9]. However, while a plethora of bioactive molecules have been proposed to either stimulate or inhibit adipogenesis and lipogenesis upon topical administration [10], no human studies have demonstrated modulation of dWAT by topical pharmacologic treatments and the simultaneous promotion of adipogenesis and lipolysis has not been explored.

To simultaneously promote adipogenesis and lipolysis, we here focused on *Kaempferia parviflora* root extract (KPRE), which has various known pharmacological activities, including anti-diabetic, anti-inflammatory and anti-diastatic effects [11,12]. KPRE had been shown to increase PPARγ expression [13], to stimulate lipolysis [14], and to decrease adipocyte size in murine 3T3-L1 cells [15]. By applying KPRE alone or in combination with long-chain fatty acids to 3-dimensional (3D) cultures of mature subcutaneous human adipocytes and differentiating primary human preadipocytes, we show that KPRE can fuel sustained preadipocyte proliferation while limiting lipogenesis and hypertrophy in mature adipocytes. Using a systems biology approach, we identify that activation of SOX9, KLF3, and GATA3 and inhibition of PPARγ, CEBPβ, CREB1 as well as SREPB1 underlie its molecular effects. We further conducted a translational study with 35 volunteers to evaluate the phenotypic impacts of topical KPRE application on facial adipose tissue fitness. Importantly, 3D imaging of facial contours with 100-μm resolution revealed significant volumetric changes with net positive volume changes in areas characterized by depletion and net negative volume changes in areas characterized by hypertrophy. In vivo volumetric analyses were confirmed in an ex vivo human skin explant model, demonstrating functional effects of KPRE on dWAT droplet size upon topical delivery. These findings thus corroborate that KPRE drives facial adipose tissue regeneration by driving preadipocyte regeneration in atrophic regions of the face while reducing lipogenesis in areas with large hypertrophic adipocytes. Combined, the presented data demonstrate that organotypic culture models of primary human adipose tissue cells can provide mechanistic insights into the molecular mechanisms of adipogenesis. The integrative utilization of in vitro, ex vivo, and clinical studies strengthens the link between molecular mechanisms and human outcomes and facilitates the rapid translation of pharmacological modulators from preclinical findings to clinical applications.

## Materials and Methods

### Botanical extracts

KPRE was supplied as a *K. parviflora* extract microemulsion (Ava Plant, Thailand), a standardized dark-brown liquid (specific gravity 1.0537; pH 5.10). The preparation is quantified by high-performance liquid chromatography and standardized against 5,7-dimethoxyflavone (DMF) as the principal marker (specification DMF >1%). The standard flavonoid profile additionally includes 5,7,4′-trimethoxyflavone and 3,5,7,3′,4′-pentamethoxyflavone at or less than 1%, with a total specific flavonoid content of >3%. We note that these values are expressed relative to the formulated microemulsion rather than to a dry extract.

### 3D culture of primary human fat cells

Organotypic 3D cultures of primary human preadipocytes and differentiated adipocytes were conducted as previously reported [16]. In short, subcutaneous stromal vascular fraction cells were cultured in flasks until they reached 70% confluency. The cells were then trypsinized and transferred to 96-well ultra-low attachment microplates (Corning) at a density of 5,000 cells per well and centrifuged at 150×*g* for 2 min. After 4 d, the culture medium was switched to a serum-free culture medium (William’s E medium supplemented with 2 mM l-glutamine, 100 U/ml penicillin, 100 μg/ml streptomycin, 10 μg/ml insulin, 10 μg/ml transferrin, 6.7 ng/ml sodium selenite, and 100 nM dexamethasone) supplemented with 500 μM 3-isobutyl-1-methylxanthine, 10 nM hydrocortisone, 2 nM 3,3′,5-triiodo-l-thyronine, 10 μM rosiglitazone, 33 μM biotin, and 17 μM pantothenic acid. After 14 d of differentiation, the spheroids were transitioned into culture medium without differentiation additives. As indicated, spheroids were treated during or after differentiation either with either of the following formulations or vehicle controls: (a) 5% (v/v) *K. parviflora* root extract alone (KPRE), (b) with a mixture of 160 μM oleic acid and 160 μM linoleic acid (SSO) conjugated with 10% bovine serum albumin at a molar ratio of 1:5, or (c) simultaneously with both KPRE and SSO (KPSS).

### Brightfield and fluorescence confocal imaging

Total spheroid area was measured from brightfield microscopy images using Fiji (ImageJ). For fluorescent microscopy, spheroids were washed in Dulbecco’s phosphate-buffered saline (PBS; Gibco), fixed for 30 min in 10% formalin (Sigma), and stained overnight protected from light using 1:2,000 BODIPY-FL (1 mg/ml; Invitrogen) and 2 μg/ml DAPI (Thermo Scientific) in PBS/0.1% Tween-20. After staining, the spheroids were washed twice with Dulbecco’s PBS and mounted on microscopy slides using an iSpacer (SunJin Lab Co., Taiwan). Imaging was performed on a LSM880 confocal microscope (Zeiss) using consistent laser settings for all samples. Measurements of lipid droplet diameter and nuclei count were carried out using a custom algorithm in Fiji (ImageJ) on stacked images.

### Expression profiling of target genes

Total RNA from adipose tissue spheroids was extracted using QIAzol Lysis Reagent (Qiagen) and the concentration of the isolated RNA was quantified using a NanoDrop spectrophotometer. The RNA was then reverse transcribed utilizing Super Script III Reverse Transcriptase (Invitrogen). Expression of target genes was analyzed by quantitative polymerase chain reaction on a 7500 Fast Real-Time PCR System (Applied Biosystems) using specific TaqMan primers (Table S1). Expression levels were calculated according to the ΔΔCt method with GAPDH as the reference gene.

### RNA sequencing

Pooled spheroids were lysed using the QIAzol lysis reagent (Qiagen), mRNA was isolated using oligo-dT magnetic beads, and samples were processed using the NEBNext Ultra II Directional RNA Library Prep Kit (NEB). Sequencing was conducted on a NovaSeq6000 instrument (Illumina) at GenomeScan BV (Leiden, Netherlands). Raw data were processed using the RTA3.4.4 pipeline and Bcl2fastq (v2.20) conversion software (Illumina). Expression levels of genes that were detected in at least 25% of samples were analyzed using Qlucore Omics Explorer v3.7 (Lund, Sweden). Reactome pathway analyses were carried out using WebGestalt [17]. The activity profiles of 500 transcription factors in adipocyte and preadipocyte spheroids were inferred for each treatment group using ISMARA [18].

### Ex vivo human skin explants

Skin tissues including the subcutaneous adipose tissue layer were obtained from a 50-year-old male donor (body mass index, 28.1; no history of metabolic diseases) and a 31-year-old female donor (body mass index, 24.4; no history of metabolic diseases). Experiments were approved by the Ethics Review Committee of Hangzhou Third People’s Hospital under permit number 2025KA184. Following collection, the skin tissue was rinsed with PBS and evenly dissected into smaller pieces using a scalpel. Each piece was placed in the upper chamber of a 12-well transwell plate (0.4 μm pore size) with the epidermis facing upward and the dermis and adipose layers facing downward. The lower chamber was filled with 2 to 3 ml of Dulbecco’s Modified Eagle Medium supplemented with 10% fetal bovine serum and 1% penicillin–streptomycin. The plate was incubated at 37 °C in a humidified atmosphere containing 5% CO_2_. After 24 h, KPSS cream or KPRE formulations (applied as a gel or as a micellized formulation) was topically applied to the epidermis once daily as indicated. The medium was refreshed every 3 d. On day 6, the tissues were collected, fixed in 4% paraformaldehyde for 24 h, dehydrated with sucrose, embedded in OCT compound, and sectioned longitudinally at a thickness of 10 μm on a microtome (LEICA 819; Shanghai Leica Instrument Co., Ltd).

### Immunohistochemistry

Frozen sections were first brought to room temperature and then rehydrated. After antigen retrieval and blocking of endogenous peroxidase activity, the sections were incubated with a primary antibody against PLIN2 (gb115593, Servicebio, Wuhan; dilution 1:200) at 4 °C overnight. Following PBS washes, the sections were incubated with a horseradish-peroxidase-conjugated secondary antibody at room temperature for 50 min. Antigen visualization was achieved using 3,3′-diaminobenzidine as chromogenic substrate, followed by counterstaining with hematoxylin. Finally, the sections were dehydrated, cleared, and mounted. All stained sections were digitally scanned using a slide scanner (Pannoramic DESK; 3DHISTECH, Shanghai) for subsequent analysis.

### Human studies

To test the effect of a composition of a topical KPSS cream, 35 adult participants between 25 and 75 years of age were prospectively included over a 10-month period between January and October 2021 in an open-label pilot imaging study. The cream consists of a water-in-oil emulsion that contains, besides the 2 active ingredients (SSO and KPRE), only penetrants, emollients, preservatives, and fragrance agents that are standard to commercial cosmetic products. Subjects were instructed to apply the composition on each side of the face from below the lower orbital rim to the jawline, excluding the forehead, commissure, and chin once or twice per day. High-resolution stereoscopic imaging and digital 3D modeling were conducted at baseline and after 3 months and 6 months of follow-up. Filler injection and facial plastic surgery were prohibited during the study and no subject received such treatment. Subjects were not paid or given other incentives to complete the study. The study was approved by an independent Institutional Review Board (IntegReview, Austin, TX; protocol number Adipeau-001) and registered at ClinicalTrials.gov under NCT04769193. All enrolled patients were included in the evaluation. Written informed consent for this study was obtained from all participants.

For 3D imaging, the participants’ faces were scanned using a Cherry Imaging 3D imaging device (Cherry Imaging, Yokneam, Israel). Imaging was guided visually and vocally by the device software (Cherry Trace). The handheld imaging device captures in real time thousands of overlapping 3D images of the face from multiple field views and angles, providing a 3D model with 100-μm resolution and accurate traceability over time. Acquisition of one hemi-face scan takes approximately 30 s. Both intra- and inter-investigator variability showed coefficients of variation of 1.8% to 3.1% and 4.4% to 7.3%, respectively, in the context of scar imaging [19]. The imaging software automatically aligns before and after images using a best-fit algorithm. Positive and negative changes in voxel height greater than the 0.5 mm are considered as changes in volume and integrated to calculate overall volumetric differences for each hemi-face model. Increases and decreases in volume were allocated to the upper, middle, and lower face using the validated assessment framework established by Flynn et al. [20], Carruthers et al. [21], and Narins et al. [22].

### Statistical analysis

Results are presented as mean ± SEM. Statistical comparisons between 2 groups were conducted using unpaired 2-tailed heteroscedastic *t* tests with *n* ≥ 3 samples per group. For human sample analyses, normality was tested using the Shapiro–Wilk test. For non-normally distributed samples, the Mann–Whitney *U* test was applied. Intra-individual changes in facial volumes over time were evaluated using paired statistical tests. For longitudinal analyses involving repeated observations from individual participants, within-subject dependence was accounted for using linear mixed-effects models with participant included as a random effect. No missing data were imputed and all data points were included without outlier removal. All statistical calculations were conducted using in Prism 9 (GraphPad, USA), R v4.02, and R Studio v2021.09.2. Given the exploratory nature of the clinical pilot study, no formal adjustment for multiple comparisons was applied, and the resulting *P* values should therefore be interpreted as exploratory. Statistical significance was established at a *P* value of ≤0.05.

## Results

### Pharmacological modulation of human preadipocyte hypertrophy and hyperplasia

**Fig. 1.**
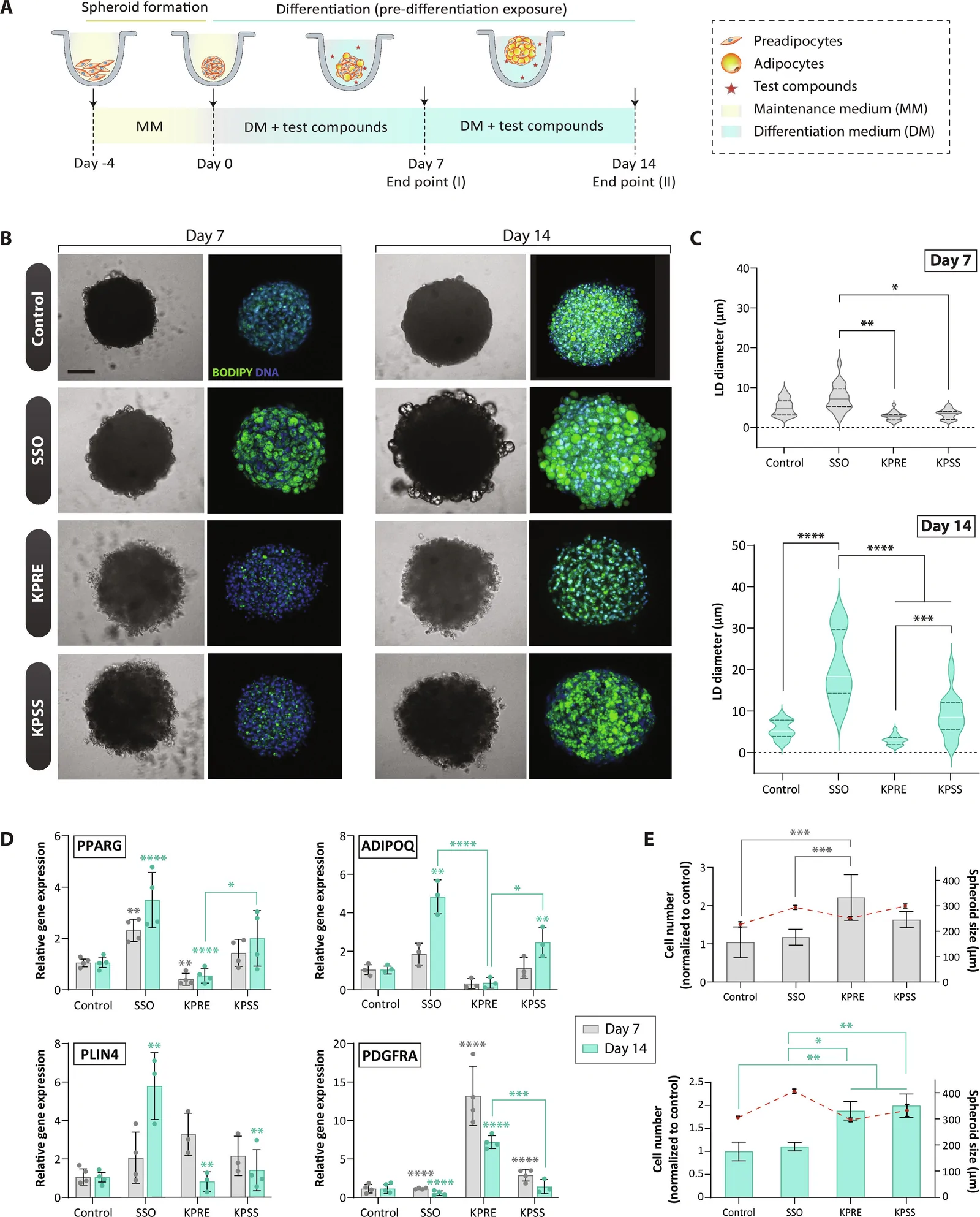
Effects of *Kaempferia parviflora* root extract (KPRE) and long-chain fatty acids on preadipocyte differentiation. (A) Schematic illustration of the experimental design. Note that 3D primary human preadipocyte spheroids are exposed to the test compounds during differentiation. (B) Phase contrast and confocal fluorescent micrographs of spheroids during differentiation while treated with different compounds. Lipid droplets are stained with BODIPY (green) and nuclei are stained with DAPI (blue). Scale bar = 100 μm. (C) Distribution of the lipid droplet (LD) diameters in adipose spheroids treated with long-chain fatty acids (SSO), KPRE, or both (KPSS) after 7 d (gray) and 14 d (green). (D) Transcriptional profile of the key adipogenic markers PPARG, ADIPOQ, and PLIN4, as well as the dedifferentiation marker PDGFRA after 7 d (gray) and 14 d (green). (E) Column plot visualizing the normalized number of nuclei in adipose spheroids under different conditions (left *y*-axis). The dashed lines indicate differences in spheroid volumes between the different groups (right *y*-axis). Note that KPRE and KPSS significantly increase cell numbers without impacting spheroid volume. *, **, ***, and **** correspond to *P* < 0.05, *P* < 0.01, *P* < 0.001, and *P* < 0.0001, respectively.

Primary adipocyte progenitors obtained from the stromal vascular fraction of human subcutaneous adipose tissue have been previously shown to efficiently differentiate in 3D culture to closely resemble mature adipocytes at the molecular level [16,23]. To investigate the molecular effects of compounds on adipogenesis, we first exposed primary human preadipocyte spheroids to SSO and KPRE during differentiation (Fig. 1A). SSO supplementation alone accelerated lipogenesis and significantly increased droplet size (7.4 ± 1.2 μm and 20.4 ± 5 μm after 7 and 14 d, respectively, compared to 4.8 ± 1.1 μm to 8.6 ± 2.1 μm in controls; Fig. 1B and C). In contrast, the formation of lipid droplets in KPRE-treated preadipocytes was delayed and droplets remained smaller than in vehicle-treated controls. Furthermore, KPRE significantly reduced the lipogenic effect of SSO throughout the culture period.

To better understand the molecular basis of these effects, we evaluated the expression of canonical mature adipocyte (PPARG, ADIPOQ, and PLIN4) and preadipocyte markers (PDGFRA). In agreement with the morphological observations, we found that SSO significantly induced expression of PPARG, ADIPOQ, and PLIN4, whereas KPRE exhibited repressive effects (Fig. 1D). Inversely, PDGFRA expression was reduced by SSO but activated >10-fold by KPRE. Combinatorial exposure (KPRE+SSO) resulted in delayed and overall lower induction of adipogenesis markers and a later repression of PDGFRA. These findings thus suggest that KPRE results in a prolonged maintenance of the undifferentiated preadipocyte state even in the presence of differentiating cues. To further evaluate the cellular consequences of this adipogenic delay, we evaluated cell numbers in spheroids after 1-week and 2-week exposures. We find that while overall spheroid volumes did not increase, cell numbers of KPRE-treated spheroids more than doubled compared to controls, whereas SSO treatment had no effect (Fig. 1E). This indicates that the maintenance of preadipocyte phenotypes allows for further rounds of mitosis, resulting in adipose microtissues with a larger number of small cells rather than fewer hypertrophic cells. Furthermore, co-treatment with KPRE and SSO (KPSS) showed the same increase in cell numbers compared to KPRE alone but with increased expression of adipogenic markers, suggesting that this condition might exert balanced effects on adipose tissue regeneration without hypertrophy.

### KPSS results in maintenance of the proliferative program in primary human preadipocytes

**Fig. 2.**
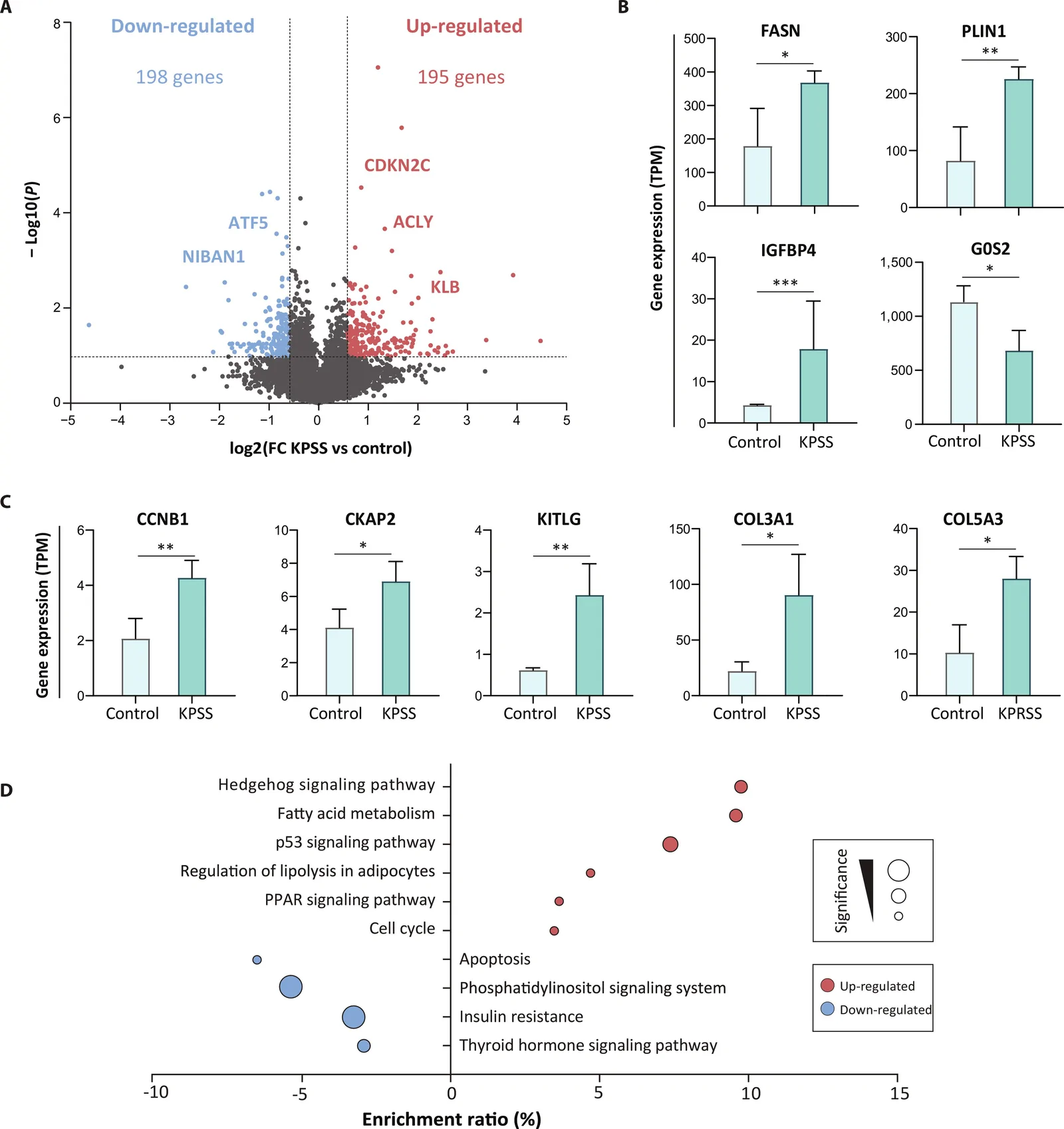
KPSS treatment of preadipocytes during differentiation promotes the prolonged maintenance of undifferentiated signatures. (A) Volcano plot showing differentially expressed genes in KPSS-treated adipose spheroids and controls after 14 d of exposure. FC, fold change. (B) KPSS impacts key factors involved in fatty acid metabolism and lipolysis. TPM, transcripts per million. (C) KPSS induces a microenvironment favoring preadipocyte maintenance and proliferation. (D) Pathway analysis of differentially expressed genes reveals that KPSS activates lipid metabolism and preadipocyte cell cycle while reducing apoptosis and insulin resistance. *, **, and *** correspond to *P* < 0.05, *P* < 0.01, and *P* < 0.001, respectively.

Given their dual effects, we investigated the molecular consequences of KPSS at first in preadipocytes using comprehensive RNA sequencing. Overall, differential gene expression analysis revealed 195 and 198 genes that were significantly up- and down-regulated, respectively (Fig. 2A). Among the most significantly up-regulated genes were the citrate lyase ACLY and fatty acid synthase (FASN) (Fig. 2B). We also found an induction of PLIN1 and down-regulation of the lipase inhibitor G0S2, suggesting that the reduced lipid content in KPRE-treated cells is at least in part due to an overall increase in lipolysis. Furthermore, β-klotho (KLB) was up-regulated >5-fold, suggesting an increase in adipocyte thermogenic responses along the FGF21 axis [24,25].

Multiple preadipocyte and dedifferentiation markers, including cell cycle factors (CCNB1 and CKAP2), factors associated with stem cell maintenance (KITLG), as well as extracellular matrix remodeling (COL3A1 and COL5A3) were significantly increased (Fig. 2C). Among the differentially up-regulated pathways, we identified the Hedgehog signaling pathway (enrichment ratio [ER] = 9.7; *P* < 0.01), fatty acid metabolism (ER = 9.6; *P* < 0.01), PPAR signaling (ER = 3.6; *P* < 0.01), and cell cycle (ER = 3.5; *P* = 0.01), whereas apoptosis (ER = −5.51; *P* = 0.02), phosphatidylinositol signaling (ER = −5.45; *P* < 0.001), and pathways related to insulin resistance (ER = −3.26; *P* < 0.001) were down-regulated (Fig. 2D).

### KPSS exerts context-specific effects depending on cellular differentiation states

**Fig. 3.**
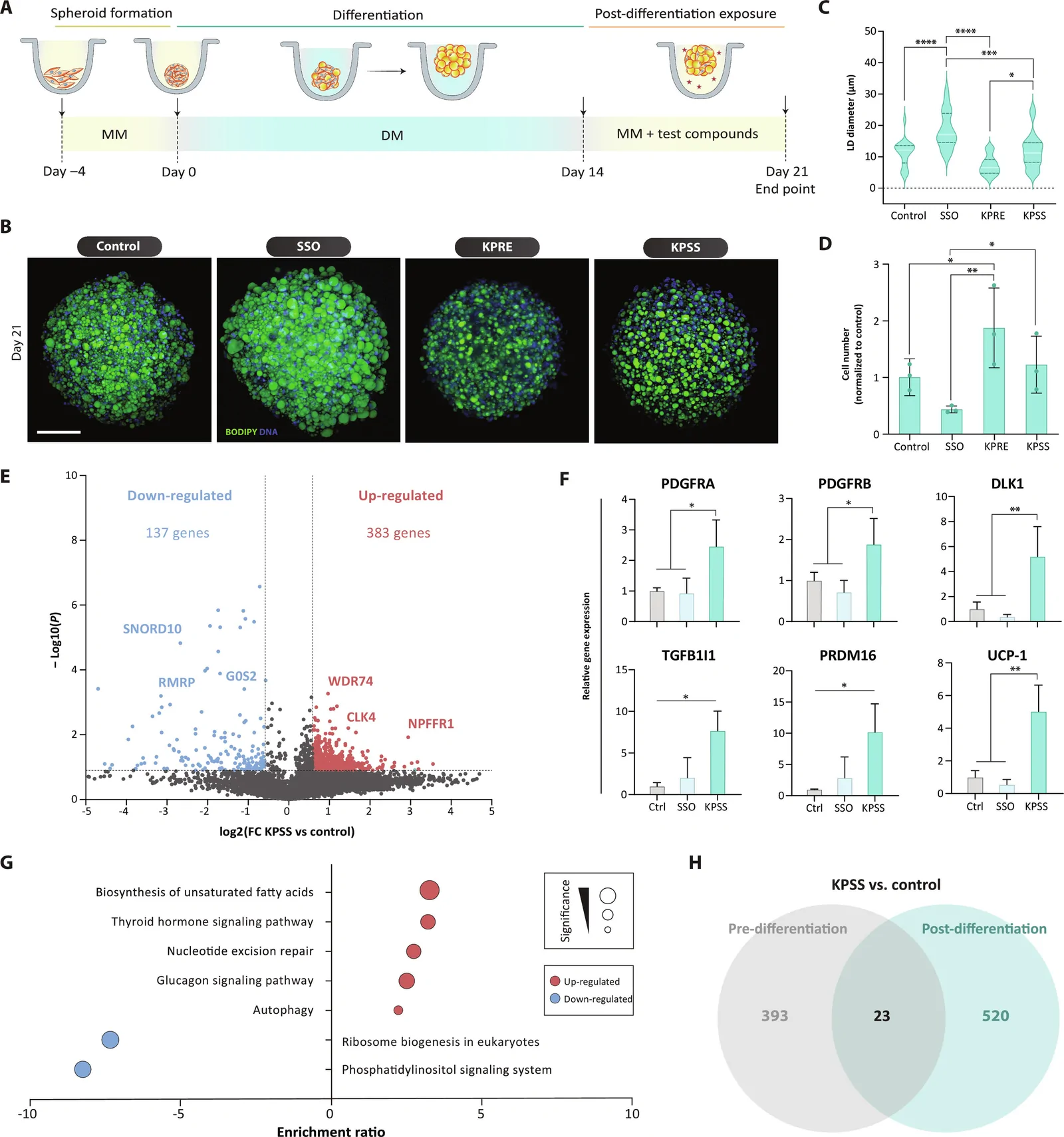
Effects of KPRE and long-chain fatty acids on differentiated human adipocytes. (A) Schematic illustration of the experimental design. Note that 3D primary human adipocyte spheroids are exposed to the test compounds after differentiation. MM, maintenance medium; DM, differentiation medium. (B) Confocal fluorescent micrographs of mature adipose spheroids treated with different compounds for 1 wk post-differentiation. Lipid droplets are stained with BODIPY (green) and nuclei are stained with DAPI (blue). Scale bar = 100 μm. (C) Distribution of the lipid droplet (LD) diameters in adipose spheroids treated with long-chain fatty acids (SSO), *Kaempferia parviflora* root extract (KPRE), or both (KPSS) for 7 d after differentiation. (D) Normalized number of nuclei in adipose spheroids treated with the test compounds for 7 d after differentiation. (E) Volcano plot showing differentially expressed genes after 7 d of exposure to KPSS post-differentiation. (F) KPSS results in elevated expression of preadipocyte markers. (G) Pathway analysis of differentially expressed genes reveals differentially regulated pathways in primary adipocytes upon KPSS exposure. (H) Venn diagram of differentially expressed genes in 3D spheroid cultures upon KPSS treatment pre- and post-differentiation. *, **, ***, and **** correspond to *P* < 0.05, *P* < 0.01, *P* < 0.001, and *P* < 0.0001, respectively.

To evaluate whether KPRE would also act on mature fat cells, we next exposed differentiated 3D human adipocyte cultures (Fig. 3A). Previous studies have demonstrated that KPRE suppresses lipid accumulation and reduces intracellular triglyceride levels in murine 3T3-L1-derived adipocyte-like cells by activating adipose triglyceride lipase and hormone-sensitive lipase independent of PPARγ transcription [15]. Consistent with these findings, we observe that KPRE reduced overall lipid accumulation and adipocyte hypertrophy in primary human adipocytes (Fig. 3B). Interestingly, KPRE appeared to impact the distribution of triglycerides in lipid droplets with most adipocytes featuring a single small lipid droplet compared to controls in which most cells were mostly pauci- or multilocular. A quantitative analysis of droplet sizes revealed that KPRE significantly reduced lipid droplet diameters (Fig. 3C). As such, the data are in agreement with the observed effects in KPRE-treated human preadipocytes during differentiation. Interestingly, KPRE also promoted the regeneration of mature adipocytes after differentiation (Fig. 3D). While mature adipocytes can reenter the cell cycle, these programs result in endoreplication and senescence rather than proliferation [26], suggesting that KPRE acts on residual populations of preadipocytes in this setting. These findings suggest that KPRE reduces the size of mature adipocytes and amplifies the propensity of preadipocytes to differentiate into mature adipocytes.

At the molecular level, we identified 137 and 383 genes that were differentially expressed upon exposure of mature adipocytes to KPSS (Fig. 3E). Among the most up-regulated genes were WDR74 and NPFFR1. WDR74 is a coactivator of TGFβ signaling, which is expressed in committed preadipocytes and is assumed to stall their differentiation by activating SMAD proteins and suppressing the expression of key transcription factors, such as PPARγ and C/EBPα [27,28]. Similarly, NPFF signaling has been shown to inhibit adipocyte differentiation [29]. We also observed increased expression of multiple bona fide markers of preadipocytes, including PDGFRA, PDGFRB, DLK1, TGFB1I1, and PRDM16 (Fig. 3F), as well as increased remodeling of the extracellular matrix (Fig. S1). Biosynthesis of unsaturated fatty acids (ER = 4.3; *P* < 0.001), thyroid hormone signaling (ER = 2.9; *P* < 0.01), nucleotide excision repair (ER = 2.8; *P* < 0.01), and glucagon signaling (ER = 2.2; *p* < 0.01) were among the most significantly altered pathways (Fig. 3G). In line with these findings, we observed an up-regulation of UCP1 and PGC1α (PPARGC1A) indicating that mitochondrial uncoupling is at least in part underlying the dissipation of stored energy associated with the reduction of lipid droplet volumes (Fig. S2A).

Interestingly, while KPSS exerted clear effects on both preadipocytes and mature adipocytes, the impacted target genes and transcriptional networks largely differed. Of the 393 and 520 genes that were differentially expressed upon KPSS exposure in preadipocytes and mature adipocytes, respectively, only 23 (~3%) were impacted in both cell types (Fig. 3H). These results indicate that KPSS activates fundamentally different signaling networks in preadipocytes and differentiated adipocytes and that these differential effects might underlie its simultaneous activation of adipocyte hyperplasia and adipogenesis.

### Comprehensive molecular profiling reveals distinct molecular networks underlying KPRE-mediated adipogenesis

**Fig. 4.**
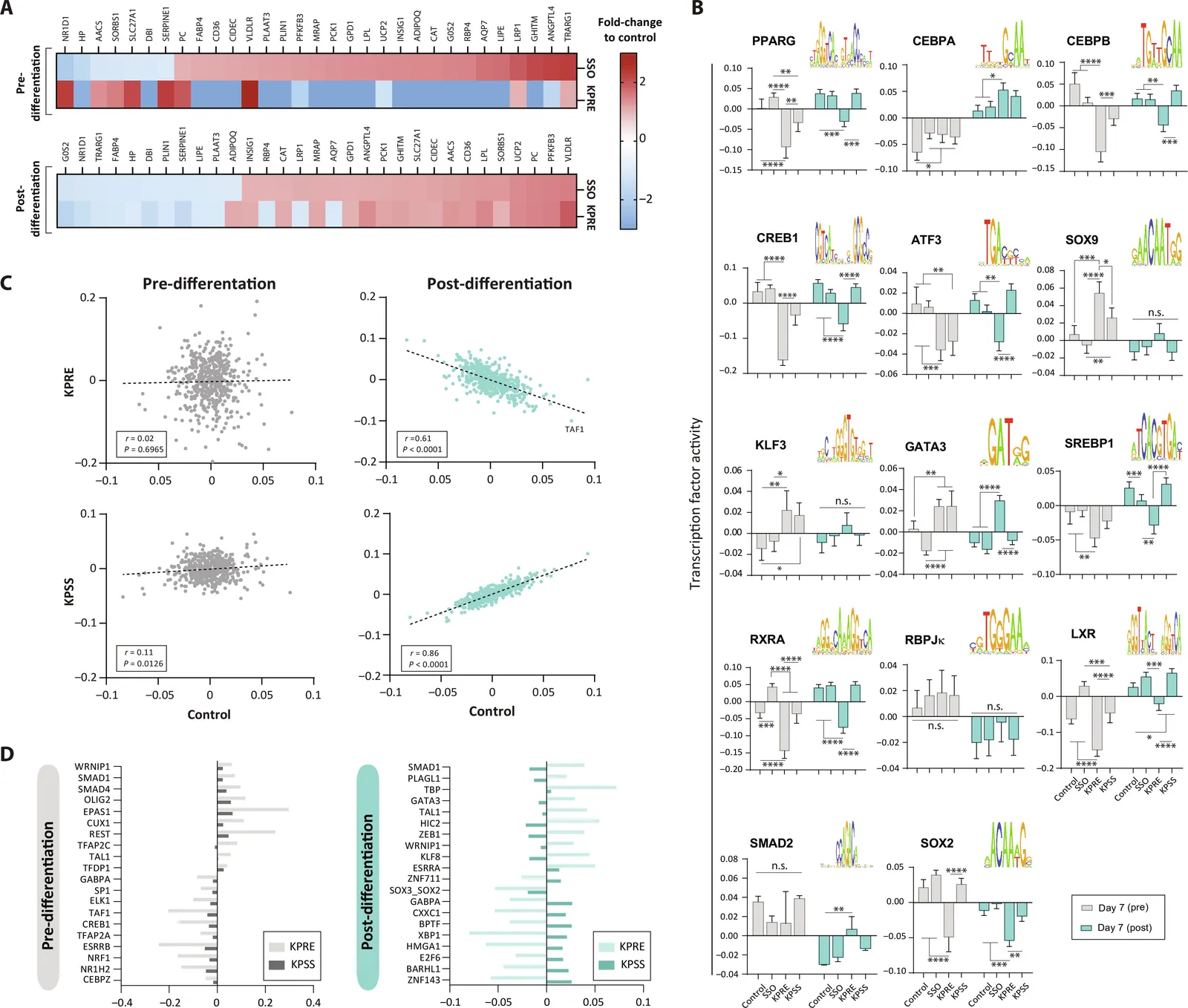
KPRE exerts differential molecular effects on preadipocytes and differentiated adipocytes. (A) KPRE and SSO have clearly distinct effects on PPARG target genes in preadipocytes (upper panel) but not in differentiated adipocytes (lower panel). (B) Activity of selected transcription factors with important roles in adipogenesis and lipogenesis are shown for preadipocytes (gray) and adipocytes after differentiation (turquoise). The binding motifs from which activity was inferred are shown for each transcription factor. *, **, ***, and **** correspond to *P* < 0.05, *P* < 0.01, *P* < 0.001, and *P* < 0.0001, respectively. n.s., not significant. (C) Scatter plots showing the correlation between changes in motif activities in KPRE- (upper row) and KPSS-treated (lower row) adipose microtissues during (left) and after (right) differentiation. (D) The top 10 transcription factors with the most increased and decreased activities are shown for adipose spheroids treated with KPRE or KPSS during (left) or after (right) differentiation.

To deconvolute transcription factor networks and identify the key drivers behind the observed transcriptomic differences, we analyzed transcription factor activities. First, we focused on PPARγ due to its critical role in adipogenesis and examined the expression of its experimentally validated target genes [30] in both pre- and post-differentiation exposure to KPRE and SSO. Notably, while SSO induced the expression of most PPARγ targets in preadipocytes, KPRE exerted opposite effects (Fig. 4A). These effects were blunted but clear also in mature adipocytes.

Next, we inferred the activities of 500 transcription factors by correlating experimentally determined binding motifs in enhancers with changes in the expression of associated genes. Interestingly, the results indicate substantial differences in the reconfiguration of transcriptional networks by KPRE, SSO, and KPSS between preadipocytes and mature adipocytes. When focusing on transcription factors with well-established roles in preadipocyte differentiation, we found that KPRE down-regulated the activity of PPARγ and CEBPβ in agreement with the observed delay in adipogenesis (Fig. 4B). Similarly, the activities of CREB1 and its target, the transcriptional repressor activating transcription factor 3 (ATF3), were reduced. In contrast, the activity of CEBPα is, if at all, increased by KPRE. Combined, these results suggest that KPRE alone inhibits the early stages of the adipocyte differentiation cascade.

Upon KPRE treatment, we observed a reduction in the activity of adipogenic transcription factors, whereas inferred motif engagement for suppressors of adipogenesis, such as SOX9 [31], KLF3 [32], and GATA3 [33], was significantly increased, particularly in preadipocytes. Besides impacting the networks underlying adipocyte differentiation, KPRE also reduced the inferred activities of the SREBP1 and RXRA, key regulators of adipocyte lipogenesis, in both preadipocytes and mature adipocytes, whereas SSO induced their activity. KPRE did not impact activity of the Notch signaling effector RBPJκ, suggesting that the observed induction of UCP1 and PGC1α does not seem to be impacted by Notch modulation. Combined, these data indicate that KPRE results in a concerted inhibition of adipogenic factors in both preadipocytes and mature adipocytes while inducing the activity of the central suppressors of preadipocyte differentiation.

Notably, co-treatment with SSO resulted in cell-type-specific effects. While KPRE’s effects on pro-adipogenic (PPARγ, CEBPβ, CREB1, and ATF3) and lipogenesis factors (SREBP1 and RXRA) as well as on adipogenesis suppressors (SOX9, KLF3, and GATA3) were ameliorated by SSO, they were still clearly maintained in preadipocytes. In contrast, in mature adipocytes, there was no significant difference between KPSS-treated adipocytes and controls with regards to the activity of these factors and only liver X receptor (LXR) activity was significantly induced. When evaluating the activities of all 500 analyzed transcription factors, we identified broad modulation of transcriptional networks resulting in an overall poor correlation of transcription factor activities between untreated and KPSS-treated cells (*r* = 0.11; Fig. 4C). In contrast, KPSS resulted in only marginal reconfiguration of transcription factor networks in differentiated adipocytes (*r* = 0.86). Strikingly, the directionality of KPRE and KPSS is similar in preadipocytes, whereas effects are mostly different in mature adipocytes (Fig. 4D). These results suggest that co-treatment with SSO modulates the cell-type-specific effects of KPRE in preadipocytes and mature adipocytes.

### Topical administration in facial atrophy and hypertrophy confirms dual adipogenic and lipolytic effects

**Fig. 5.**
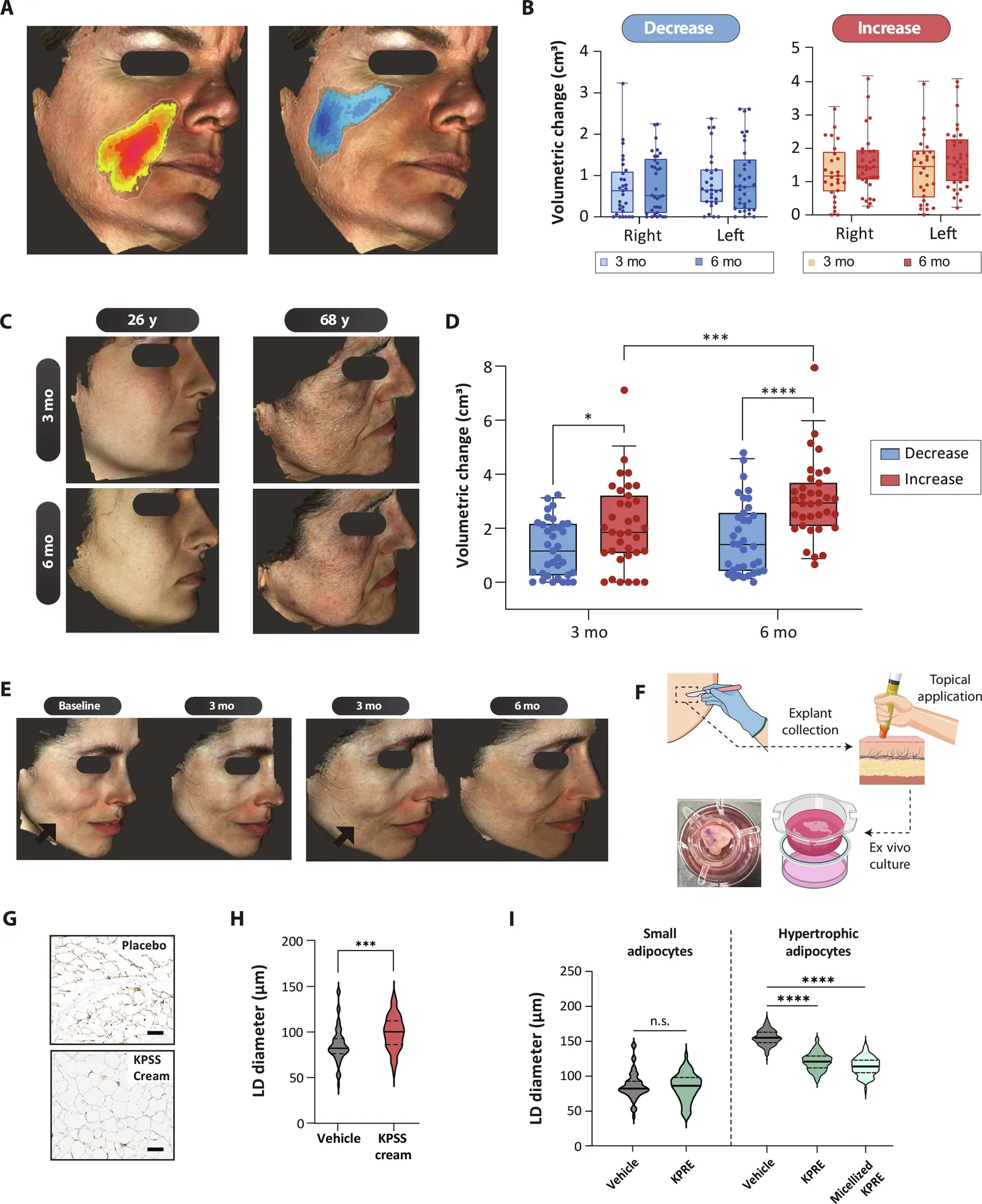
Topical application of KPRE cream results in coordinated modulation of facial adipose tissue volume in vivo and ex vivo. (A) Representative high-resolution stereoscopic 3D images of changes in facial adipose tissue volume showing nonoverlapping increases (red shades) and decreases in volume (blue shades), jointly contributing to improved facial symmetry. (B) Facial areas of decreased and increased volumes are shown for each subject. (C) 3D images of the right hemi-face of the youngest (26 y) and oldest (68 y) participant are shown after 3 mo and 6 mo of treatment. (D) The combined volumetric changes are shown for the entire course of the treatment period. Note that areas of increased volumes (red) continue to increase over the course of 6 mo, whereas volume decreases occur only throughout the first 3 mo. (E) Representative images depicting changes in one of the participants from baseline up to 6 mo. Arrows indicate areas of atrophy. (F) Schematic illustration of the ex vivo experiments. (G) PLIN2 immunohistochemistry of ex vivo full-thickness human skin explants treated for 6 d under different experimental conditions. Scale bar = 100 μm. (H) Quantification of lipid droplet diameter in the dermal adipose layer, demonstrating significant enlargement upon treatment with KPSS cream. (I) Topical treatment of ex vivo human skin explants with a KPRE formulation. Note that no effect of droplet size is observed in small adipocytes, whereas a significant reduction is seen in explants with hypertrophic adipocytes. *, ***, and **** correspond to *P* < 0.05, *P* < 0.001, and *P* < 0.0001, respectively; n.s., not significant.

To evaluate the translational potential of our mechanistic findings, we conducted a pilot single-site open-label human study using a topical KPSS cream containing both KPRE and SSO. Specifically, we performed high-resolution stereoscopic 3D imaging of facial volumes in 35 participants (4 males and 31 females; mean age 46.5 ± 10.5 y) at baseline and following topical application of KPSS emulsion for 3 and 6 months. Strikingly, contemporaneous increases and decreases in facial volume were observed in different areas of the face in the same individual (Fig. 5A). At 6 months, the median volume increase was 1.48 cm^3^ for the left hemi-face and 1.39 cm^3^ for the right hemi-face, while the median decrease was 0.72 and 0.52 cm^3^ for the left and right hemi-face, respectively (Fig. 5B; Table S2). Older participants showed a greater increase in facial volume than younger participants, independent of treatment duration with an incremental increase in volume of 0.43 cm^3^ per 10 years of subject age (*P* = 0.04; Fig. 5C). In contrast, there was no significant association of age with volume decreases (*P* = 0.1). Longitudinal analyses revealed that increases in volume of both hemi-face measurements combined were significantly greater at 6 months than at 3 months (*P* = 0.03), whereas the decreases in volume were not significantly different (*P* = 0.73; Fig. 5D). In the linear mixed model, every month of use was associated with an incremental combined increase in volume of 0.18 cm^3^ (*P* = 0.04), corresponding to a progressive volumetric increase in regions classified as atrophic alongside volume reductions in regions classified as hypertrophic (Fig. 5E).

Notably, this volumetric gain in hypotrophic regions was recapitulated at the cellular level in an ex vivo human skin explant model (Fig. 5F). In this system, topical application of the KPSS cream for 6 d resulted in a significant enlargement of dermal adipocyte lipid droplets, as shown by PLIN2 immunohistochemistry and quantitative image analysis (Fig. 5G and H). To furthermore directly test the effect of KPRE, we treated explants with adipocytes of different sizes with topical formulations containing only the KPRE and excipients but only minimal levels of long-chain fatty acids (Fig. 5I). In these experiments, KPRE exhibited no significant effect on the size of lipid droplets in small adipocytes; however, in hypertrophic adipocytes a highly significant reduction in droplet diameter was observed. These effects were strongest when a micellized formulation was used, in agreement with their reported increased penetration of the outer layer of the skin and augmented bioavailability [34].

Together, these results provide evidence that the active substances in KPRE-containing formulations can access and modulate dermal adipose tissue following topical application. Furthermore, the concordance in directionality of in vivo volumetric remodeling and the ex vivo and in vitro adipocyte responses support a shared underlying mechanism. Thus, the observed increase in volume in atrophic regions and decrease in hypertrophic regions provide preliminary human evidence supporting the region-specific dual effects predicted by our organotypic models. These findings thus highlight the value of an integrated use of in vitro and ex vivo human tissue models for the translation of mechanistic insights at the molecular and cellular level toward evaluation of measurable outcomes in humans.

## Discussion

There is growing appreciation that adipocyte regeneration declines with age. In the face, insufficient regeneration of dermal fat cells leads to hollowing, as well as hypertrophy-induced changes in skin strength. While long-chain fatty acids enhance preadipocyte maturation, they also promote hypertrophy in mature adipocytes. The results presented here demonstrate that co-treatment with long-chain fatty acids (SSO) and KPRE can uncouple these opposing effects, offering a novel approach to address age-related facial adipose changes. These findings are made possible by an integrated translational workflow that comprises in vitro primary human adipocyte spheroids, ex vivo full-thickness human skin models, and in vivo human volumetric imaging to elucidate and validate the dual adipogenic and lipolytic actions of KPRE.

**Fig. 6.**
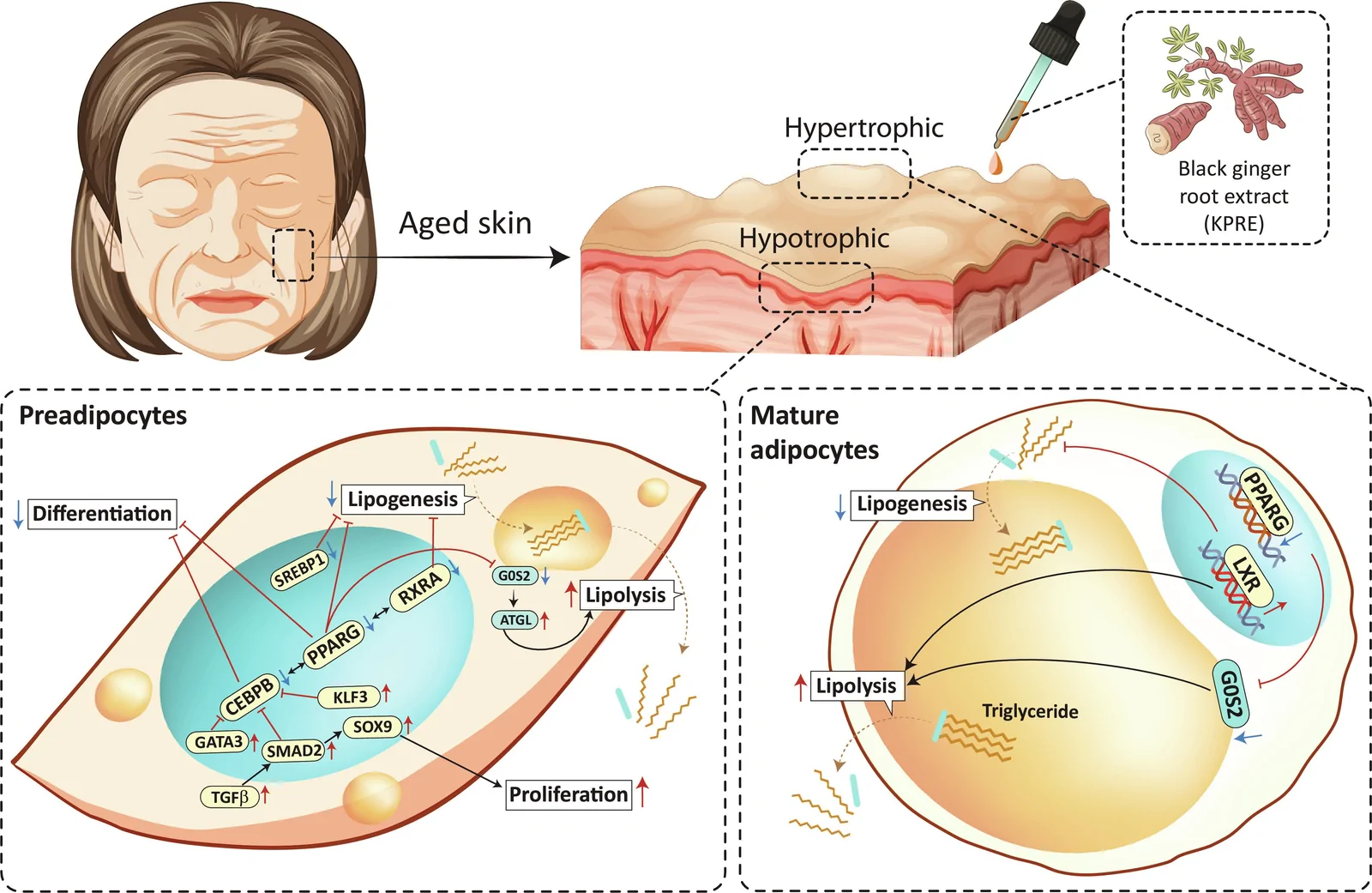
Proposed mechanistic model of the differentiation state-dependent effects of KPRE in human adipose cells. In preadipocytes, transcriptional and transcription factor activity analyses suggest modulation of the TGFβ–SMAD2–SOX9 axis together with a stimulation of GATA3 and KLF3 and inhibition of PPARγ activity. Based on their established roles in adipocyte biology, these changes provide a candidate regulatory framework for the delayed differentiation and sustained proliferation observed in KPRE-treated preadipocytes. In mature adipocytes, KPRE treatment is associated with reduced PPARγ activity and G0S2 expression, as well as modulation of liver X receptor (LXR) activity. Together with the observed reduction in adipocyte size, these molecular changes are consistent with enhanced lipolysis and reduced lipogenesis. The depicted pathways represent a proposed mechanistic model based on the integration of phenotypic, transcriptomic, and transcription factor activity data. However, further functional validation by targeted genetic or pharmacological experiments is required to establish the causality of individual factors.

Mechanistic in vitro assays in preadipocytes revealed that KPRE was effective at mitigating the lipogenic effects of SSO, resulting in significantly smaller lipid droplet sizes. These effects are likely to be driven by increased lipolysis as evidenced by KPRE-mediated down-regulation of G0S2, which directly inhibits the rate-limiting lipolytic enzyme adipose triglyceride lipase [35], as well as reduced lipogenesis due to an inhibition of RXRA and SREBP1 [36,37]. Furthermore, KPSS also saw sustained regeneration with cell numbers increasing throughout the treatment period. At the molecular level, KPRE treatment is associated with increased inferred motif activities of SOX9, KLF3, and GATA3, which are known repressors of the PPARγ–C/EBPβ network that controls adipogenesis [31,33,38]. This effect seems to be at least in part due to an activation of the TGFβ–SMAD signaling axis, which has been shown to repress C/EBP transactivation [39]. This results in the prolonged maintenance of an undifferentiated preadipocyte state as well as in the activation of proliferative programs resulting in preadipocyte expansion. Jointly, these data thus suggest that KPRE has multifaceted effects on preadipocytes, resulting in the prolonged maintenance of preadipocyte identities, activation of preadipocyte regenerative programs, and mitigation of long-chain-fatty-acid-driven hypertrophy (Fig. 6A).

KPRE also impacted the phenotypes of mature adipocytes. Post differentiation, KPRE reduced mature adipocyte size and, in combination with SSO, was similarly effective at mitigating the lipogenic effects of fatty acid supplementation. However, unlike in the pre-differentiation setting where cell numbers in the KPSS group were higher than in controls, the addition of KPRE reduced the suppressive effect of SSO on regeneration but did not increase the overall proliferation rate. At the molecular level, KPRE alone exerted mostly similar effects on mature adipocytes including the inhibition of PPARγ and C/EBPβ as well as of SREBP1 and RXRA. However, the preadipocyte factors KLF3 and SOX9 were not significantly induced in mature adipocytes, consistent with the inability of mature adipocytes to regress toward a preadipocyte state. The most striking difference between the 2 settings was that co-treatment with SSO ameliorated the KPRE effects at the molecular level in differentiated adipocytes. While several transcription factors were significantly affected by KPSS in preadipocytes, only LXR activity was altered in mature adipocytes. LXR has complex roles in adipose tissue [40]. Specifically, LXR induces basal lipolysis in human adipocytes [41] and seems to repress lipogenesis [42], although recent data indicate that the latter might be context dependent [43]. Together, these findings are consistent with a model in which the phenotypic effects observed in KPSS-treated adipocytes involves increased lipolysis and reduced lipogenesis (Fig. 6B). However, further functional validation using direct measurements of glycerol or free fatty acid release or lipase activity would be required to directly demonstrate an increase in lipolytic flux.

To facilitate the translation of preclinical results, it is important to consider mechanisms of topical delivery that are compatible with the requirements of the facial adipose tissue. The dermal white adipose tissue contains populations of mature adipocytes and preadipocytes with vellus and terminal hair follicles extending into its upper layers. The pores surrounding hair follicles provide a potential pathway for targeting this layer of adipocytes. The trans-follicular route is generally accessible to molecules smaller than 500 Da with the necessary lipophilic properties, whereas larger molecules cannot pass the corneal layer [44]. The bioactive constituents in KPRE are presumed to be polymethoxyflavonoids, with molecular weights ranging from 268 to 372 Da. The skin permeability of 3 representative polymethoxyflavonoids, PMF (3,5,7,3′,4′-pentamethoxyflavone), DMF (5,7-dimethoxyflavone), and TMF (5,7,4′-trimethoxyflavone), was previously evaluated in porcine skin [45]. All 3 compounds were found to penetrate the skin effectively, paving the way for the clinical application of KPSS formulations as topical modulators of adipose tissue regeneration. Rapid phenotypic changes seen upon topical application of KPSS cream in ex vivo human skin explants corroborated these observations, jointly indicating that bioactive KPRE constituents can be effectively delivered to the subcutaneous adipose tissue layer also in human skin.

In vivo high-resolution stereoscopic imaging of human volunteers confirmed a distinct volumetric pattern in human facial tissue, with balanced gains and reductions in facial volume. Based on the mechanism of action of the KPSS formulation, we hypothesize that the increases in volume occurred by stimulation of adipogenesis that exceeded any lipolytic effects in hypotrophic areas of the face; conversely, decreases in volume occurred where the lipolytic effects exceeded any adipogenic activity in areas of hypertrophy. This is to the best of our knowledge the first study that reports a dual mechanism of action that is selectively controlled by the different local adipose tissue microenvironments. These volumetric patterns were mirrored at the cellular level in the ex vivo skin model, where KPSS cream enlarged dermal adipocyte lipid droplets in a manner consistent with the adipogenic component of the in vivo gains. This concordance between model systems highlights the translational value of combining mechanistic tissue platforms with human 3D imaging to accelerate the path from molecular insight to real-world application.

Notably, volumetric gains and losses behave differently over time. Gains are progressive, increasing significantly between 3 and 6 months, whereas losses plateau after the first 3 months. Furthermore, gains scale with participant age, while losses show no significant age association. We interpret the progressive, age-dependent nature of the gains as consistent with restoration of deficient adipocyte number through adipogenesis. Because adipogenic capacity declines with age, older faces may carry a larger reservoir of unmet adipogenic potential, which the formulation could re-engage. In contrast, progressive lipogenic hypertrophy would be expected to produce a different temporal and cellular signature than the one we observe. We nonetheless note that a lipogenic contribution cannot be excluded in some individuals, particularly those with higher baseline adiposity. The plateau in volume losses is consistent with a self-limiting process; if lipolytic activity is more permissive in hypertrophic adipocytes which are limited in number, this finite substrate approaches a floor. A portion of the apparent regional volume reduction may additionally reflect contour redistribution, whereby restoration of volume in atrophic regions reduces the relative prominence of adjacent fullness or laxity; such a geometric contribution would likewise be inherently self-limiting. We would like to emphasize that these attempts at explaining the temporal and regional differences are hypotheses motivated by the observed data rather than as established mechanisms and note that direct discrimination among them will require longer-term and histologically resolved studies.

There were notable limitations to the pilot in vivo evaluation. Skin elasticity, which was defined as a primary endpoint, could not be determined in a way that was representative of the entire face. This decision was made early in the study on methodological grounds before any outcome data were analyzed. The study moreover did not meet our recruitment goal of 75 patients due to the slow recruitment process during the COVID-19 pandemic, but we confirm that all recruited patients were included in the analysis and no outlier removal was performed. The clinical component of this study should be interpreted in the context of its exploratory design. Specifically, the study was open-label and non-randomized with a relatively small cohort spanning a broad age range. Consequently, the observed volumetric changes cannot be attributed exclusively to the intervention, and contributions from normal biological variation, changes in hydration, body weight or dermal structure, lifestyle-related factors, or imaging variability cannot be excluded. While the concordance between the clinical observations and the effects predicted by the organotypic models is encouraging, the clinical findings should be validated in adequately powered, randomized, placebo-controlled studies to establish clinical efficacy and determine the robustness of the region-specific effects observed here. Furthermore, the ex vivo skin explant experiments were conducted using tissues from only 2 donors; validation across a larger and more diverse donor cohort will be important to assess interindividual variability and confirm the generalizability of the observed effects. Additionally, while integration of transcriptomic profiling, transcription factor activity inference, and phenotypic assays identifies candidate regulatory pathways underlying the effects of KPRE, their causal contributions remain to be established through targeted genetic or pharmacological perturbation studies.

Combined, our data provide a successful translational example for the application of 3D primary human tissue cultures to mechanistically deconvolute the effects of therapeutic formulations on adipose tissue phenotypes. At the molecular level, we show that KPRE and long-chain-fatty-acid formulations exert dual context-specific effects that delay adipogenesis and promote human preadipocyte maintenance while stimulating lipolysis in mature adipocytes. In vivo, this results in the regeneration of subcutaneous adipose tissue in atrophic areas and the coordinated reversal of compensatory hypertrophy, providing a novel mechanism for facial adipose rejuvenation.

## Data Availability

All relevant data to support the conclusions are included in the paper. Underlying raw data are, where legally possible, available from the corresponding authors upon request.

## References

[1] Swift A, Liew S, Weinkle S, Garcia JK, Silberberg MB. The facial aging process from the “inside out”. Aesthet Surg J. 2020;41(10):1107–1119. 10.1093/asj/sjaa339 PMC8438644 33325497

[2] Galanin I, Nicu C, Tower JI. Facial fat fitness: A new paradigm to understand facial aging and aesthetics. Aesth Plast Surg. 2021;45(1):151–163. 10.1007/s00266-020-01933-6 32914326

[3] Yang C, Zhang P, Xing X. Tear trough and palpebromalar groove in young versus elderly adults. Plast Reconstr Surg. 2013;132(4):796–808. 24076672 10.1097/PRS.0b0133182a0539e

[4] Sullivan PK, Hoy EA, Mehan V, Singer DP. An anatomical evaluation and surgical approach to the perioral mound in facial rejuvenation. Plast Reconstr Surg. 2010;126(4):1333–1340. 20885255 10.1097/PRS.0b013e3181ea4abd

[5] Abboushi N, Yezhelyev M, Symbas J, Nahai F. Facelift complications and the risk of venous thromboembolism: A single center’s experience. Aesthet Surg J. 2012;32(4):413–420. 22446820 10.1177/1090820X12442213

[6] Nicu C, Pople J, Bonsell L, Bhogal R, Ansell DM, Paus R. A guide to studying human dermal adipocytes in situ. Exp Dermatol. 2018;27(6):589–602. 29603400 10.1111/exd.13549

[7] Widgerow AD, Kilmer SL, Garruto JA, Stevens WG. Non-surgical fat reduction and topical modulation of adipose tissue physiology. J Drugs Dermatol. 2019;18(4):375–380. 31012732

[8] Yanting C, Yang QY, Ma GL, Du M, Harrison JH, Block E. Dose- and type-dependent effects of long-chain fatty acids on adipogenesis and lipogenesis of bovine adipocytes. J Dairy Sci. 2018;101(2):1601–1615. 29153512 10.3168/jds.2017-13312

[9] Zhang Z, Shao M, Hepler C, Zi Z, Zhao S, An YA, Zhu Y, Ghaben AL, Wang MY, Li N, et al. Dermal adipose tissue has high plasticity and undergoes reversible dedifferentiation in mice. J Clin Invest. 2019;129(12):5327–5342. 31503545 10.1172/JCI130239 PMC6877341

[10] Moseti D, Regassa A, Kim W-K. Molecular regulation of adipogenesis and potential anti-adipogenic bioactive molecules. Int J Mol Sci. 2016;17(1):124. 26797605 10.3390/ijms17010124 PMC4730365

[11] Kusirisin W, Srichairatanakool S, Lerttrakarnnon P, Lailerd N, Suttajit M, Jaikang C, Chaiyasut C. Antioxidative activity, polyphenolic content and anti-glycation effect of some Thai medicinal plants traditionally used in diabetic patients. Med Chem. 2009;5(2):139–147. 19275712 10.2174/157340609787582918

[12] Sae-Wong C, Matsuda H, Tewtrakul S, Tansakul P, Nakamura S, Nomura Y, Yoshikawa M. Suppressive effects of methoxyflavonoids isolated from *Kaempferia parviflora* on inducible nitric oxide synthase (iNOS) expression in RAW 264.7 cells. J Ethnopharmacol. 2011;136(3):488–495. 21251970 10.1016/j.jep.2011.01.013

[13] Horikawa T, Shimada T, Okabe Y, Kinoshita K, Koyama K, Miyamoto KI, Ichinose K, Takahashi K, Aburada M. Polymethoxyflavonoids from *Kaempferia parviflora* induce adipogenesis on 3T3-L1 preadipocytes by regulating transcription factors at an early stage of differentiation. Biol Pharm Bull. 2012;35(5):686. 22687402 10.1248/bpb.35.686

[14] Park SH, Park J, Lee M, Kim J, Eun S, Jun W, Kim OK, Lee J. Antiobesity effect of *Kaempferia parviflora* accompanied by inhibition of lipogenesis and stimulation of lipolysis. Food Nutr Res. 2023;3:67. 10.29219/fnr.v67.9374 PMC10335092 37441513

[15] Okabe Y, Shimada T, Horikawa T, Kinoshita K, Koyama K, Ichinose K, Aburada M, Takahashi K. Suppression of adipocyte hypertrophy by polymethoxyflavonoids isolated from *Kaempferia parviflora*. Phytomedicine. 2014;21(6):800–806. 24629599 10.1016/j.phymed.2014.01.014

[16] Shen JX, Couchet M, Dufau J, de Castro Barbosa T, Ulbrich MH, Helmstädter M, Kemas AM, Zandi Shafagh R, Marques MA, Hansen JB, et al. 3D adipose tissue culture links the organotypic microenvironment to improved adipogenesis. Adv Sci. 2021;8(16):2100106. 10.1002/advs.202100106 PMC8373086 34165908

[17] Liao Y, Wang J, Jaehnig EJ, Shi Z, Zhang B. WebGestalt 2019: Gene set analysis toolkit with revamped UIs and APIs. Nucleic Acids Res. 2019;47(W1):W199–W205. 31114916 10.1093/nar/gkz401 PMC6602449

[18] Balwierz PJ, Pachkov M, Arnold P, Gruber AJ, Zavolan M, Van Nimwegen E. ISMARA: Automated modeling of genomic signals as a democracy of regulatory motifs. Genome Res. 2014;24(5):869–884. 24515121 10.1101/gr.169508.113 PMC4009616

[19] Hendel K, Ortner VK, Fuchs CSK, Eckhouse V, Haedersdal M. Dermatologic scar assessment with stereoscopic imaging and digital three-dimensional models: A validation study. Lasers Surg Med. 2021;53(8):1043–1049. 33389766 10.1002/lsm.23373

[20] Flynn TC, Carruthers A, Carruthers J, Geister TL, Görtelmeyer R, Hardas B, Himmrich S, Kerscher M, de Maio M, Mohrmann C, et al. Validated assessment scales for the upper face. Dermatologic Surg. 2012;38:309–319. 10.1111/j.1524-4725.2011.02248.x 22316187

[21] Carruthers J, Flynn TC, Geister TL, Görtelmeyer R, Hardas B, Himmrich S, Jones D, Kerscher M, De Maio M, Mohrmann C, et al. Validated assessment scales for the mid face. Dermatologic Surg. 2012;38:320–332. 10.1111/j.1524-4725.2011.02251.x 22316188

[22] Narins RS, Carruthers J, Flynn TC, Geister TL, Görtelmeyer R, Hardas B, Himmrich S, Jones D, Kerscher M, De Maio M, et al. Validated assessment scales for the lower face. Dermatologic Surg. 2012;38:333–342. 10.1111/j.1524-4725.2011.02247.x 22316189

[23] Ioannidou A, Alatar S, Schipper R, Baganha F, Åhlander M, Hornell A, Fisher RM, Hagberg CE. Hypertrophied human adipocyte spheroids as in vitro model of weight gain and adipose tissue dysfunction. J Physiol. 2021;600(4):869–883. 34387376 10.1113/JP281445

[24] Samms RJ, Cheng CC, Kharitonenkov A, Gimeno RE, Adams AC. Overexpression of β-klotho in adipose tissue sensitizes male mice to endogenous FGF21 and provides protection from diet-induced obesity. Endocrinology. 2016;157(4):1467–1480. 26901091 10.1210/en.2015-1722

[25] Moure R, Cairo M, Moron-Ros S, Quesada-Lopez T, Campderros L, Cereijo R, Hernáez A, Villarroya F, Giralt M. Levels of β-klotho determine the thermogenic responsiveness of adipose tissues: Involvement of the autocrine action of FGF21. Am J PhysiolEndocrinol Metab. 2021;320(4):E822–E834. 10.1152/ajpendo.00270.2020 33615874

[26] Li Q, Hagberg CE, Silva Cascales H, Lang S, Hyvönen MT, Salehzadeh F, Chen P, Alexandersson I, Terezaki E, Harms MJ, et al. Obesity and hyperinsulinemia drive adipocytes to activate a cell cycle program and senesce. Nat Med. 2021;27(11):1941–1953. 34608330 10.1038/s41591-021-01501-8

[27] Liu J, Zhao M, Yuan B, Gu S, Zheng M, Zou J, Jin J, Liu T, Feng XH. WDR74 functions as a novel coactivator in TGFβ signaling. J Genet Genomics. 2018;45(12):639–650. 30594465 10.1016/j.jgg.2018.08.005

[28] Hepler C, Shan B, Zhang Q, Henry GH, Shao M, Vishvanath L, Ghaben AL, Mobley AB, Strand D, Hon GC, et al. Identification of functionally distinct fibro-inflammatory and adipogenic stromal subpopulations in visceral adipose tissue of adult mice. Elife. 2018;7: Article e39636. 30265241 10.7554/eLife.39636 PMC6167054

[29] Lefrère I, De Coppet P, Camelin JC, Le Lay S, Mercier N, Elshourbagy N, Bril A, Berrebi-Bertrand I, Feve B, Krief S. Neuropeptide AF and FF modulation of adipocyte metabolism. Primary insights from functional genomics and effects on beta-adrenergic responsiveness. J Biol Chem. 2002;277(42):39169–39178. 12149260 10.1074/jbc.M205084200

[30] Fang L, Zhang M, Li Y, Liu Y, Cui Q, Wang N. PPARgene: A database of experimentally verified and computationally predicted PPAR target genes. PPAR Res. 2016;2016(1):6042162. 27148361 10.1155/2016/6042162 PMC4842375

[31] Gulyaeva O, Nguyen H, Sambeat A, Heydari K, Sul HS. Sox9-Meis1 inactivation is required for adipogenesis, advancing Pref-1+ to PDGFRα+ cells. Cell Rep. 2018;25(4):1002–1017.e4. 30355480 10.1016/j.celrep.2018.09.086 PMC6903418

[32] Sue N, Jack BH, Eaton SA, Pearson RC, Funnell AP, Turner J, Czolij R, Denyer G, Bao S, Molero-Navajas JC, et al. Targeted disruption of the basic Krüppel-like factor gene (Klf3) reveals a role in adipogenesis. Mol Cell Biol. 2008;28(12):3967–3978. 18391014 10.1128/MCB.01942-07 PMC2423134

[33] Tong Q, Tsai J, Tan G, Dalgin G, Hotamisligil GS. Interaction between GATA and the C/EBP family of transcription factors is critical in GATA-mediated suppression of adipocyte differentiation. Mol Cell Biol. 2005;25(2):706–715. 15632071 10.1128/MCB.25.2.706-715.2005 PMC543409

[34] Parra A, Jarak I, Santos A, Veiga F, Figueiras A. Polymeric micelles: A promising pathway for dermal drug delivery. Materials. 2021;14(23):7278. 34885432 10.3390/ma14237278 PMC8658125

[35] Yang X, Lu X, Lombès M, Rha GB, Chi YI, Guerin TM, Smart EJ, Liu J. The G0/G1 switch gene 2 regulates adipose lipolysis through association with adipose triglyceride lipase. Cell Metab. 2010;11(3):194–205. 20197052 10.1016/j.cmet.2010.02.003 PMC3658843

[36] Metzger D, Imai T, Jiang M, Takukawa R, Desvergne B, Wahli W, Chambon P. Functional role of RXRs and PPARγ in mature adipocytes. Prostaglandins Leukot Essent Fat Acids. 2005;73(1):51–58. 10.1016/j.plefa.2005.04.007 15936932

[37] Crewe C, Zhu Y, Paschoal VA, Joffin N, Ghaben AL, Gordillo R, Oh DY, Liang G, Horton JD, Scherer PE. SREBP-regulated adipocyte lipogenesis is dependent on substrate availability and redox modulation of mTORC1. JCI Insight. 2019;4(15): Article e129397. 10.1172/jci.insight.129397 PMC6693888 31310592

[38] Wu Z, Wang S. Role of kruppel-like transcription factors in adipogenesis. Dev Biol. 2013;373(2):235–243. 23142072 10.1016/j.ydbio.2012.10.031

[39] Choy L, Derynck R. Transforming growth factor-β inhibits adipocyte differentiation by Smad3 interacting with CCAAT/enhancer-binding protein (C/EBP) and repressing C/EBP transactivation function. J Biol Chem. 2003;278(11):9609–9619. 12524424 10.1074/jbc.M212259200

[40] Laurencikiene J, Rydén M. Liver X receptors and fat cell metabolism. Int J Obes. 2012;36(12):1494–1502. 10.1038/ijo.2012.21 PMC3520012 22370853

[41] Stenson BM, Ryden M, Venteclef N, Dahlman I, Pettersson AM, Mairal A, Åström G, Blomqvist L, Wang V, Jocken JW, et al. Liver X receptor (LXR) regulates human adipocyte lipolysis. J Biol Chem. 2011;286(1):370–379. 21030586 10.1074/jbc.M110.179499 PMC3012995

[42] Korach-André M, Archer A, Gabbi C, Barros RP, Pedrelli M, Steffensen KR, Pettersson AT, Laurencikiene J, Parini P, Gustafsson JÅ. Liver X receptors regulate de novo lipogenesis in a tissue-specific manner in C57BL/6 female mice. Am J Physiol Endocrinol Metab. 2011;301(1):E210–E222. 21521718 10.1152/ajpendo.00541.2010

[43] Kleiboeker B, He A, Tan M, Lu D, Hu D, Liu X, Goodarzi P, Hsu FF, Razani B, Semenkovich CF, et al. Adipose tissue peroxisomal lipid synthesis orchestrates obesity and insulin resistance through LXR-dependent lipogenesis. Mol Metab. 2024;82: Article 101913. 38458567 10.1016/j.molmet.2024.101913 PMC10950804

[44] Bos JD, Meinardi MMHM. The 500 Dalton rule for the skin penetration of chemical compounds and drugs. Exp Dermatol. 2000;9(3):165–169. 10839713 10.1034/j.1600-0625.2000.009003165.x

[45] Tuntiyasawasdikul S, Limpongsa E, Jaipakdee N, Sripanidkulchai B. Transdermal permeation of *Kaempferia parviflora* methoxyflavones from isopropyl myristate-based vehicles. AAPS PharmSciTech. 2014;15(4):947–955. 24789664 10.1208/s12249-014-0122-y PMC4113614

