# Supplementary material for: Dual Modulation of Adipogenesis and Lipolysis Augments Facial Adipose Tissue Restoration
Source: Comput Struct Biotechnol J. 2026 Sep 14;35(1):0213. doi: 10.34133/csbj.0213 (PMC13572798; doi:10.34133/csbj.0213)
Supplement: Supplementary file 1 [file csbj.0213.f1.pdf]

Supplementary Table 1 - Specific TaqMan primers used for gene expression analysis.

Gene	Species	TaqMan Probe ID
GAPDH	Human	hs99999905_m1
ADIPOQ	Human	hs00605917_m1
PPARG	Human	hs00234592_m1
PDGFRA	Human	hs00998018_m1
PLIN4	Human	hs00287411_m1

Supplementary Table 2 - Six-month volume increases (↑) and decreases (↓) in cubic centimeters (cc).

Subject	Age (years)	Right ↑ (cc)	Left ↑ (cc)	Right ↓ (cc)	Left ↓ (cc)
1	46	NA	2.09	1.91	2.61
2	32	0.49	2.41	0	0.33
3	31	1.59	1.71	1.47	1.00
4	32	1.09	2.40	0.67	0.00
5	44	NA	3.29	NA	1.33
6	57	1.45	0.98	0.48	0.72
7	56	1.39	0.63	0.49	0.91
8	57	1.45	0.98	0.48	0.72
9	52	1.46	2.37	1.22	1.54
10	52	0.45	0.21	1.17	0.55
11	57	1.88	1.79	2.23	2.56
12	42	1.45	2.71	1.54	0.74
13	49	0.48	1.48	1.62	0.78
14	49	0.99	2.18	1.05	2.07
15	51	1.65	1.26	0.00	0.66
16	46	1.64	1.71	0.23	0.31
17	55	1.14	4.00	0.51	0.07
18	32	1.16	1.44	0.08	0.17
19	40	1.91	1.14	0.24	2.05
20	35	1.43	1.06	0.24	2.05
21	35	2.83	1.22	0.05	0.13
22	54	1.92	2.21	1.3	2.61
23	36	1.14	0.84	1.2	0.75
24	48	2.91	2.58	1.47	1.85
25	48	0.42	0.51	0.00	0.00
26	26	0.24	0.75	0.00	0.43
27	64	1.98	0.43	1.37	0.17
28	47	1.36	1.73	1.60	0.96
29	49	0.91	1.60	0.03	0.14
30	60	3.55	1.37	0.10	0.20
31	38	1.24	1.28	2.24	1.15
32	68	4.09	3.85	0.16	1.26
33	43	1.07	0.85	0.52	0.23
34	35	1.12	1.82	0.00	0.38
35	63	2.14	0.99	0.44	0.00

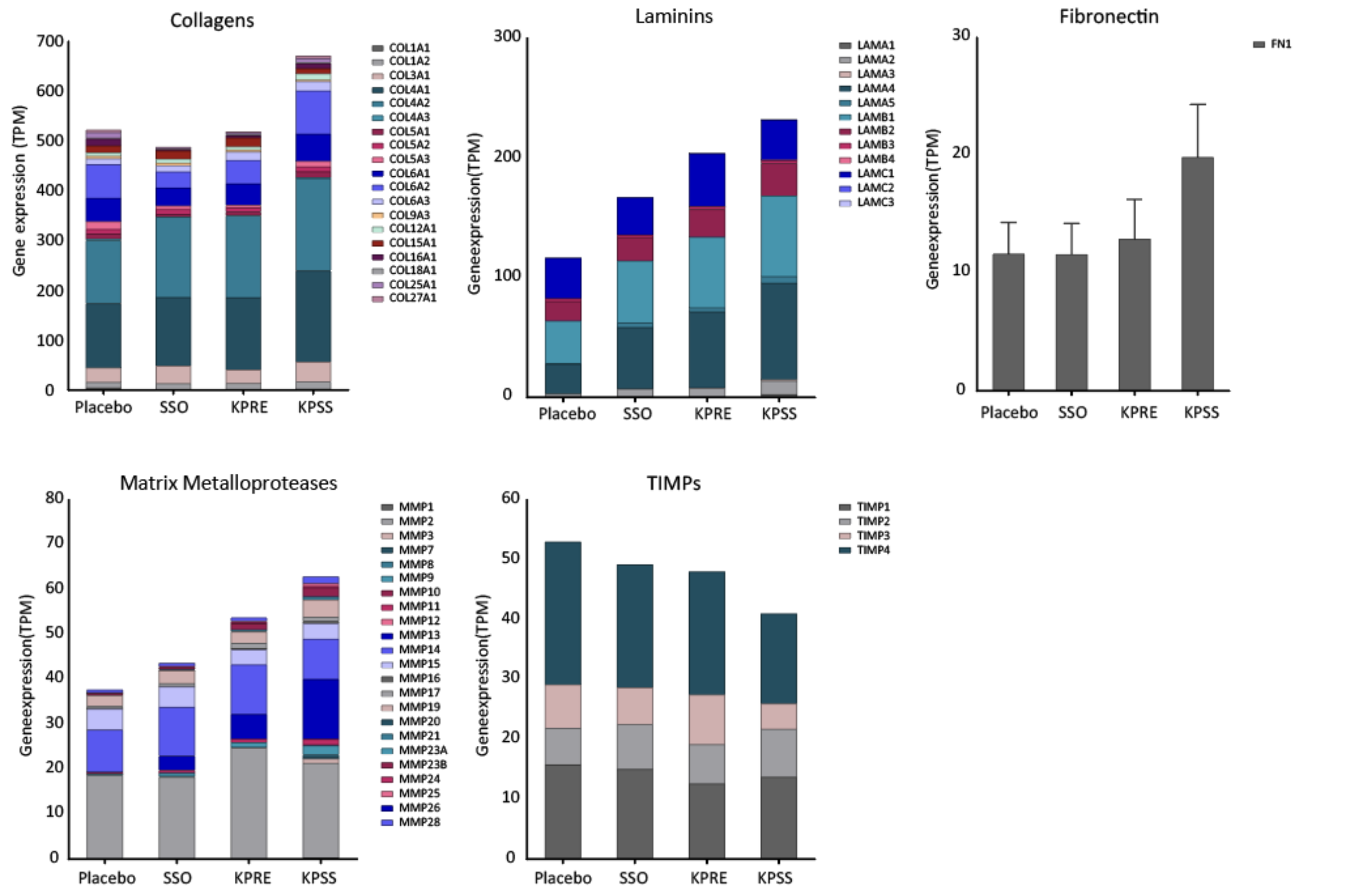

Supplementary Figure 1 - Expression profiling of structural ECM proteins (Collagens, fibronectin, and laminins) and ECM remodeler families (MMPs and TIMPs) in post-differentiation groups.

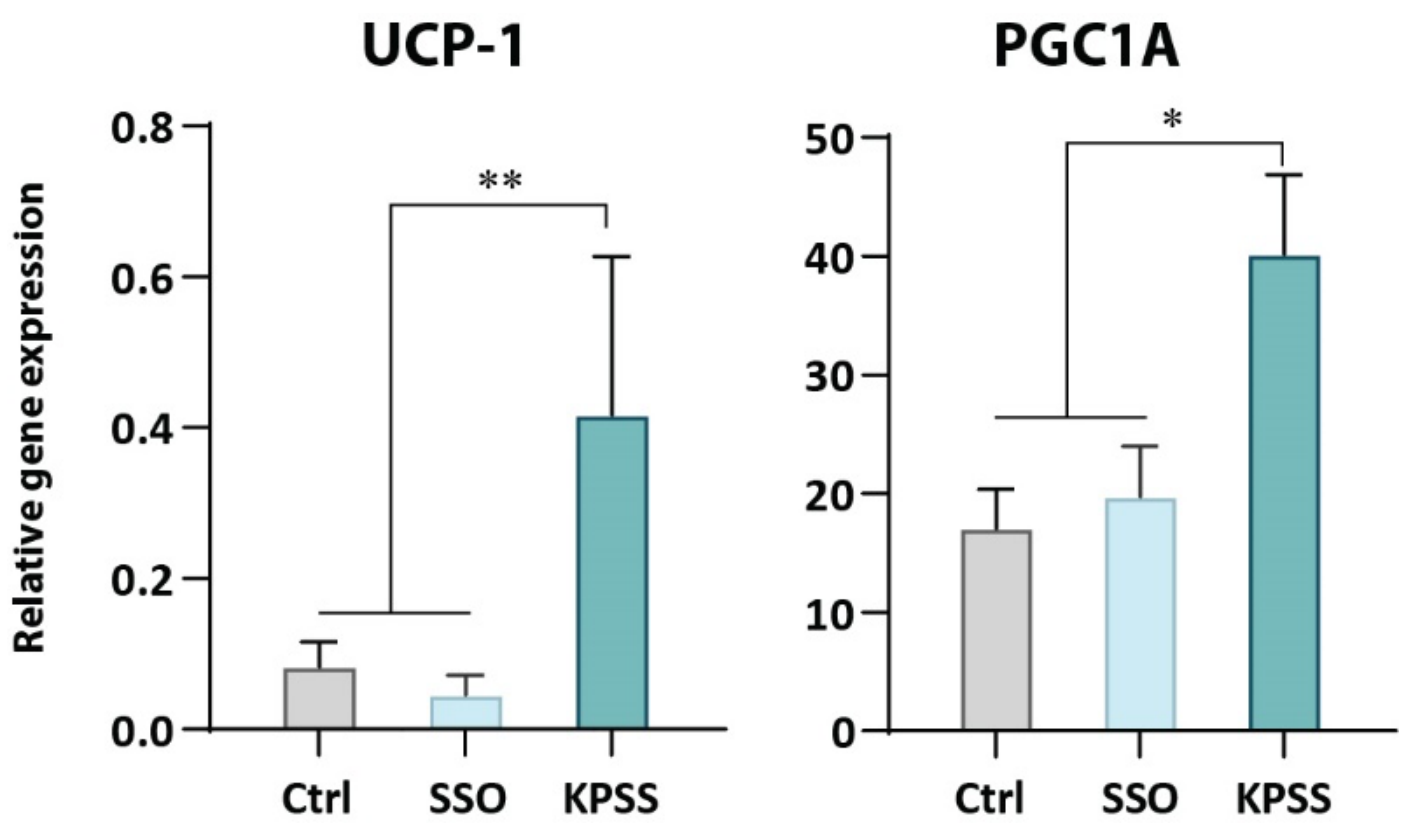

Supplementary Figure 2 - Expression of UCP-1 and PGC1A among different treatment groups. * and ** indicates $p<0.05$ and $p<0.01$ in unpaired two-tailed heteroscedastic t-tests.
